# Correlations between canine allantoamniotic fluid and fetal development throughout the pregnancy term

**DOI:** 10.3389/fvets.2025.1686014

**Published:** 2025-10-15

**Authors:** Phakawat Tantitaveewattana, Narong Tiptanavattana, Thitsana Ingkasri, Angkhana Dermlim, Thanawat Ampornpong, Artima Uppathamchat, Peruch Jirayuwattanakun

**Affiliations:** ^1^Faculty of Veterinary Science, Prince of Songkla University, Songkhla, Thailand; ^2^District Livestock Office, Singha Nakhon District Office, Songkhla, Thailand; ^3^Public Health and Environment Division, Trang City Municipality, Trang, Thailand

**Keywords:** dog, fetal parameter, ultrasonography, allantoamniotic fluid depth, pregnancy

## Abstract

Canine gestation typically lasts 63–65 days after the LH surge. Although ultrasonography is well established for monitoring fetal development in dogs, little is known about the dynamics of amniotic fluid. This study aimed to evaluate allantoamniotic fluid (AAF) using ultrasonographic examinations and correlate it with various factors of normal fetal development. Pregnant American Bulldogs underwent ultrasonographic examination (from weeks 3 to 8), and fetal parameters were collected weekly from 29 fetuses (*n* = 29). This study determined values for allantoamniotic fluid by cross-sectional view (AAFD_C_), allantoamniotic fluid by longitudinal view (AAFD_L_), biparietal diameter (BPD), cardio-thoracic ratio (CTR), abdominal cross-sectional area (ACA), gastric area (GA), and intercostal space (ICS). The correlation between fetal parameters and gestational age was analyzed. The AAFD_C_ and AAFD_L_ were first detected at week 3. Their maximum values, 34.60 ± 7.89 mm and 33.51 ± 9.59 mm, respectively, were observed at week 6 (*p* < 0.05). Subsequently, these values significantly and steadily declined from week 7 to the end (*p* < 0.05). In contrast, the BPD, ACA, GA, and ICS increased significantly from week 8 and were highly significantly correlated with gestation (*p* < 0.0001). Moreover, AAFD_C_ and AAFD_L_ showed a moderate correlation with day of gestation (DG) and day before parturition (DBP) (*p* < 0.0001). In conclusion, these findings propose the establishment of AAFD_C_ and AAFD_L_ as novel parameters for evaluating fetal wellbeing in canine practice.

## 1 Introduction

In humans, the routine monitoring of fetal development is standard, with practical guidelines for perinatal pregnancy. However, the clear guidelines for the monitoring of normal fetal development are not yet well-established in dogs. Previous studies in dogs have been reported primarily through radiography and ultrasonography with parturition predictors and important indicators, e.g., visible organ development, size of fetal organs, fetal heartbeats, and fetal movement (1). Assessing fetal health in puppies presents challenges and considerations by gestational age and breed-specific characteristics. There is a direct impact on the evaluation and detection of fetal maturity and development. Recently, there have been limited studies on this topic (2–4). The gestational period of a dog normally ranges from 57 to 72 days after breeding (1, 5–7). Radiography and ultrasonography are routinely used for pregnancy monitoring and parturition prediction. However, these techniques have been used in the case of (1) the first detection of fetal organs to estimate gestational day (such as gestational sac, placenta, stomach, kidney, eyes, and gastrointestinal tract) (1, 4, 8–10) and (2) the determination of parturition date by formulating the size of extra-embryonic and extra-fetal structures [such as inner chorionic cavity (ICC), placental thickness, and outer uterine diameter (OUD)] or fetal structures [such as the size of crown rump length (CRL), biparietal diameter (BPD), body diameter, deep portion of diencephalo-telencephalic vesicle (DPTV), and kidney diameter] (8, 11–15).

In human antenatal care, assessing congenital abnormalities is also critically important beyond estimating gestational age and predicting parturition date. For example, assessment of amniotic fluid (AF) appearance and volume (16, 17), which is a standard parameter, can assist in evaluating the risk of specific abnormalities, such as oligohydramnios, which can indicate urinary tract obstruction, dysfunctional kidneys, abnormal placental function, and neonatal intensive care admission (18, 19). In addition, the volume and characteristics of amniotic fluid reflect the important aspects of fetal development and maturity (16, 20, 21).

For pregnant dogs, precisely recognizing fetal maturity is also key to planning the delivery date and ensuring viable, healthy neonates (22). However, estimating fetal development in dogs is complicated by interbreed variability, which limits the universal applicability of existing parameters. Previous studies have speculated that the allantoamniotic fluid (AAF) may provide important insights into neonatal maturity in dogs, as in humans, where scientific findings have already been established (22, 23). While the veterinary literature on AAF is limited, it predominantly addresses other species (24, 25). Consequently, the potential diagnostic role of amniotic fluid in dogs remains largely unknown (26, 27). To assess the volume of AF with non-invasive techniques, AF index measurements and the single deepest vertical pocket (SDVP) have been well established for assessment during pregnancy (28, 29). However, this AF assessment protocol has not yet been studied in dogs. Therefore, this study is the first to non-invasively evaluate the patterns and interrelationships of AAF with other fetal parameters. This study proposes these findings as complementary diagnostic markers. These markers can effectively challenge the interbreed variability in canine perinatal evaluation.

To date, no study has reported on the ultrasonographic assessment of AAF during fetal development throughout pregnancy in dogs. Therefore, the primary objective of this study was to describe the developmental changes of various fetal parameters (AAFD_C_, AAFD_L_, BPD, CTR, ACA, GA, and ICS) in normal puppies using ultrasonographic measurements. A secondary objective was to determine the correlation between these parameters and day of gestation (DG) and day before parturition (DBP), as well as their interrelationships. We, therefore, hypothesized that these parameters are positively correlated with gestational age and that a strong interrelationship exists among them, reflecting a synchronized and predictable growth pattern.

## 2 Materials and methods

This study was ethically performed according to the Prince of Songkla University Animal Care and Use Protocol (accession no. 2563–05-041). A G-power analysis was performed to calculate the minimum number of fetuses that should be included for this study. G-power analysis showed that the minimum number of animals needed was 29, considering an r value of 0.5, an alpha value (two-tailed) of 0.05, and a power of 0.8. The formulation of the total sample size is [(Z_α_ + Z_β_)/C]^2^ + 3. The fetal measurements were obtained from the fetuses (*n* = 29) by transabdominal ultrasonography. Five female American bulldogs, aged 2–4 years old and weighing between 20 and 25 kilograms, were included in this study. The litter size ranged from 5 to 8 puppies per dam. Before breeding, all dogs were routinely examined for health status and breeding soundness examinations. All puppies included in the study were required to be healthy and free from congenital disorders, particularly those affecting the cardiovascular, digestive, and urinary systems, as well as any other disorders leading to neonatal death.

### 2.1 Study designs

The mating plan used for breeding in this study was adapted from the protocol described in (9, 30). First, the timing of ovulation of the study was determined by a combination of serial vaginal cytology and serial progesterone assays, a widely accepted method for accurate breeding management (30–32). Each female dog was monitored for ovulation time by measuring serum progesterone levels using Bionote’s Vcheck V200 analyzer (Bionote Inc., Hwaseong, South Korea). Artificial insemination (AI) was then carried out using fresh semen. Ultrasonographic examination was performed weekly from days 21 (week 3) to 62 (week 9) after ovulation to confirm pregnancy and collect fetal parameters (3, 4, 8, 9). The date of Cesarean section was determined by both presentation of labor signs (e.g., excitement, dropped core body temperature, or clear fluid from the vagina) and a decrease in serum progesterone level, which was not greater than 4.00 ng/mL (33).

### 2.2 Sonographic measurements

**Figure 1:**
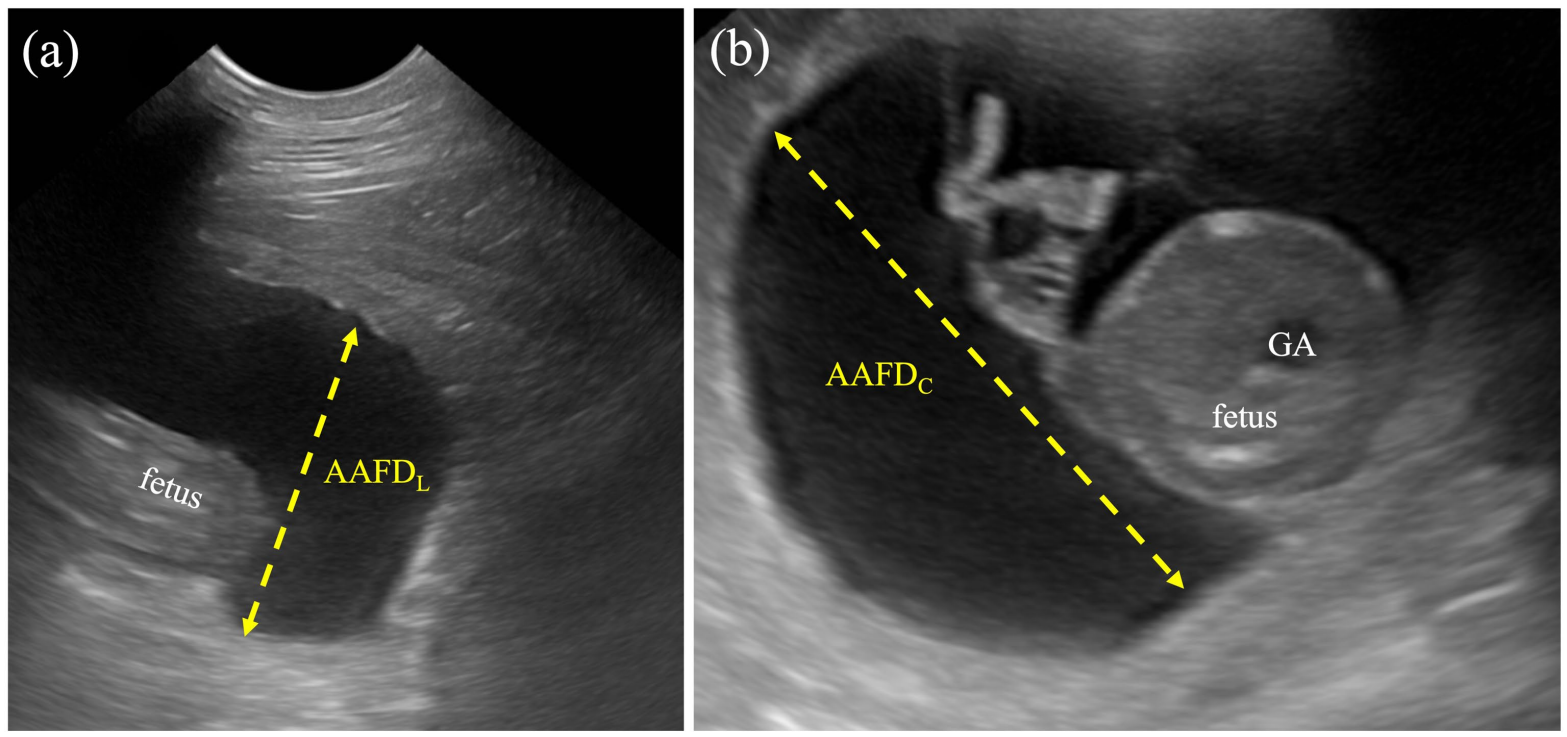
Measurement protocol for AAFD_L_ and AAFD_C_. **(a)** The AAFD_L_ was measured as the deepest sac at the caudal regions of the fetus, from inner edge to inner edge, excluding fetal structures. **(b)** The AAFD_C_ was measured in the deepest region of the fetal trunk, specifically at the widest part of the cross-sectional view of the fetus.

**Figure 2:**
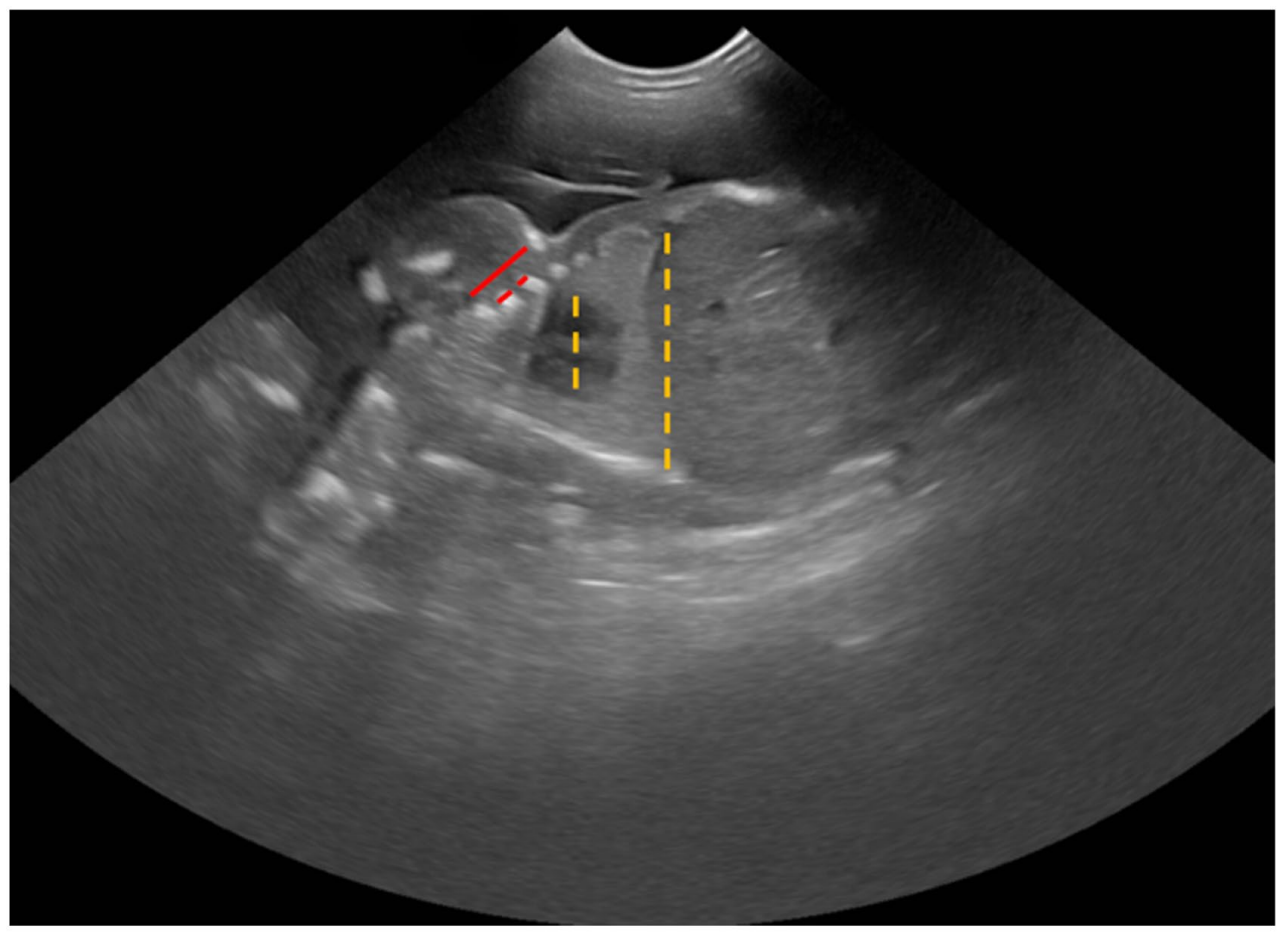
Sonographic measurement of CTR (dashed line) and ICS (solid line).

Dogs underwent ultrasonographic examination in dorsal recumbency and were performed without sedation or anesthesia using transabdominal ultrasonography (Mindray DC-80 X-Insight, Shenzhen Mindray Bio-Medical Electronics Co., Ltd., Shenzhen, China) with a 4.0–10.0 MHz micro-convex probe for the diagnosis of pregnancy and fetal measurements. All dogs generally underwent sonographic evaluation with hair clipping and the use of acoustic coupling gel on the ventral abdomen. All fetal parameters were weekly performed and marked the position of the fetus, and then ultrasonographic images were measured by using SYNAPSE® software version 4.4.1 (Fujifilm Medical Systems, NC, USA). Three trained observers independently performed the ultrasonographic examinations and measurements, and the mean value of each parameter was used in this study. Fetal measurements were calculated as follows:

1. Allantoamniotic fluid volume (AAFV) was evaluated by using both allantoamniotic fluid depth by cross-sectional view (AAFD_C_) and longitudinal view (AAFD_L_) without the fetus’s presentation. AAFD_C_ was an average of the two deepest regions at the fetal trunk, whereas the AAFD_L_ was an average of the deepest sac (inner-to-inner edges) at the cranial and caudal regions (Figure 1). These values were adapted from single deepest pocket (SDP) and deepest vertical pocket (DVP) measurements in humans (34, 35).
2. BPD was measured from the distance between both sides of the parietal bone (outer-to-outer edge) in a symmetrically cross-sectional sonograph.
3. Cardio-thoracic ratio (CTR) was calculated by measuring the largest distance at the middle level of the fetal heart (outer-to-outer edge) and then dividing by the longest distance at the middle level of the last rib (Figure 2). Both measurements were done in a dorsal plane sonograph.
4. Abdominal cross-s.ectional area (ACA) was calculated from the two largest perpendicular truncal wall diameters (outer-to-outer edges) in a cross-sectional sonograph.
5. Gastric area (GA) was calculated from the largest cross-sectional stomach diameter in two perpendicular axes (outer-to-outer edges).

Both ACA and GA were calculated by the equation of an ellipse (*π*/4 *A*B; A = diameter from the outer-to-outer edge of the truncal wall or stomach, and B = diameter from the perpendicular axis).

1. Intercostal space (ICS) was determined by measuring the distance from the caudal edge of the first rib to the cranial edge of the fourth rib, subtracting the diameters of the second and third ribs, and then dividing that total by three (Figure 2).

### 2.3 Statistical analysis

All data in this study were expressed as mean and standard deviation, and the statistical analysis was performed using GraphPad Prism (version 9.5.0 (730) GraphPad Software, San Diego, USA). Comparisons between values of fetal parameters from different weeks were analyzed by ANOVA. Pearson’s Square was applied to analyze the coefficient of determination (r^2^) between fetal parameters. The level of significance was set at a *p*-value of <0.05. Linear regression between gestational age, which included DG and DBP, and fetal parameters, was performed and presented as y = a + bx equation form (y = gestational age, a = intercept of the coefficient, and b = first-order coefficient).

## 3 Results

### 3.1 Descriptive analysis of allantoamniotic fluid (AAF) by ultrasonographic examinations

**Figure 3:**
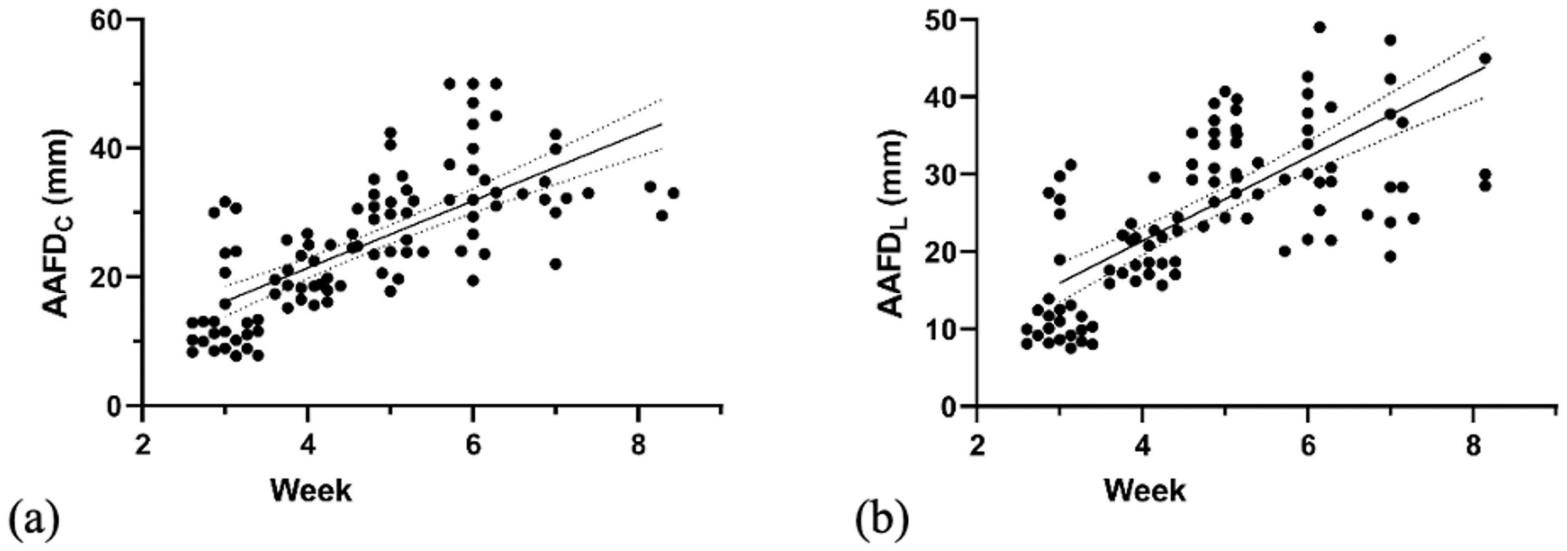
Linear regression graph illustrates the correlation of **(a)** AAFD_C_ (weeks 3–8) and **(b)** AAFD_L_ (weeks 3–8) with gestational weeks.

To determine the depth of AAF, the deepest region between the inner edges of the embryonic sac was measured. The AAFD_C_ and AAFD_L_ were first detected at week 3 with mean values of 14.71 ± 7.49 mm and 14.13 ± 7.57 mm, respectively. Maximum AAFD_C_ and AAFD_L_ were measured at week 6 as 34.60 ± 7.89 and 33.22 ± 5.75 mm, respectively (*p* < 0.05) (Figure 3; Table 1). Subsequently, both parameters significantly and steadily declined from week 7 to the end (*p* < 0.05).

**Table 1:** Mean values with standard deviation (x̄±SD) of AAFD_C_ and AAFD_L_ of gestational weeks.

Fetal parameters	Gestational week (period)					
	Week 3 (21–27)	Week 4 (28–34)	Week 5 (35–41)	Week 6 (42–48)	Week 7 (49–55)	Week 8 (56–62)
AAFD_C_ (mm)	14.71 ±7.49^a^	20.52 ± 3.76^b^	28.97 ± 6.45^c^	34.60 ± 7.89^d^	33.22 ± 5.75^c,d^	32.19 ± 2.38^c,d^
AAFD_L_ (mm)	14.13 ±7.57^a^	20.10 ± 3.49 ^a^	32.08 ± 5.11^b^	33.51 ± 9.59 ^b^	31.22 ± 10.94^b^	40.38 ± 13.91^b^

^a–d^ Values within the same row with the same superscript letter are not statistically different (*p* > 0.05), while those with different letters are significantly different (*p* < 0.05). Fetal parameters from different weeks were analyzed by ANOVA.

### 3.2 Descriptive analysis of other fetal parameters

**Figure 4:**
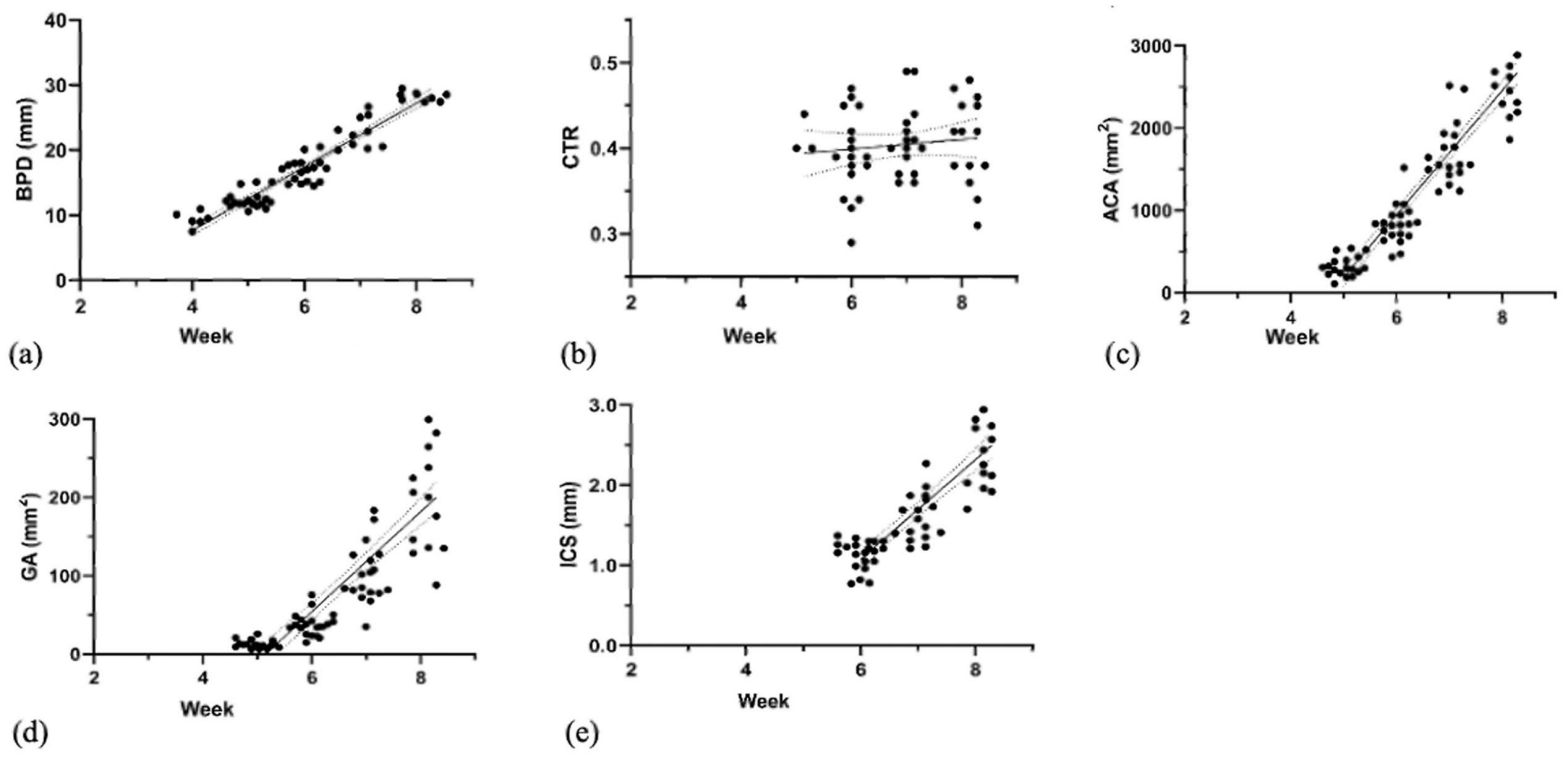
Linear regression graph illustrates the correlation of **(a)** BPD (weeks 4–8), **(b)** CTR (weeks 5–8), **(c)** ACA (weeks 5–8), **(d)** GA (weeks 5–8), and **(e)** ICS (weeks 6–8) with gestational weeks.

This part describes the normal fetal development of the head, thorax, and abdomen sections. This study examined the developmental changes in fetal parameters (BPD, CTR, ACA, GA, and ICS) across gestational weeks (Figure 4 and Table 2). Continued growth was observed in most parameters. Specifically, BPD increased significantly from weeks 4 to 8 (*p* < 0.0001). Similarly, GA, ACA, and ICS also showed significant increases from weeks 6 to 8 (*p* < 0.0001). In contrast, the CTR showed steady values throughout the measurement period (*p* > 0.05).

**Table 2:** Mean values with standard deviation (x̄±SD) of other fetal parameters (BPD, CTR, ACA, GA, and ICS) of gestational weeks.

Fetal parameters	Gestational week (period)					
	Week 3 (21–27)	Week 4 (28–34)	Week 5 (35–41)	Week 6 (42–48)	Week 7 (49–55)	Week 8 (56–62)
BPD (mm)	-	9.36 ± 1.16^a^	12.39 ± 1.33^b^	16.93 ± 1.78^c^	23.70 ± 3.14^d^	28.16 ± 0.76^e^
CTR	-	-	0.47 ± 0.07^a^	0.44 ± 0.05^a^	0.45 ± 0.05^a^	0.44 ± 0.06^a^
ACA (mm^2^)	-	-	321.71 ± 122.24^a^	829.49 ± 237.87^b^	1781.45 ± 451.12^c^	2638.95 ± 479.04^d^
GA (mm^2^)	-	-	12.66 ± 5.37^a^	37.97 ± 14.31^a^	111.18 ± 45.55^b^	214.82 ± 78.71^c^
ICS (mm)	-	-	-	1.14 ± 0.18^a^	1.63 ± 0.29^b^	2.54 ± 0.44^c^

^a-e^ Values within the same row with the same superscript letter are not statistically different (*P* > 0.05), while those with different letters are significantly different (*p* < 0.05). Fetal parameters from different weeks were analyzed by ANOVA. Not applicable data (depends on the fetal structure development).

### 3.3 Correlation of fetal parameters with day of gestation (DG) and day before parturition (DBP)

This study investigated the relationship between fetal parameters (BPD, ACA, ICS, GA, AAFD_C,_ and AAFD_L_) and both the day of gestation (DG) and the day before parturition (DBP) (Table 3). Our analysis revealed that BPD, ACA, ICS, and GA were highly significantly correlated (*p* < 0.0001) with DG and DBP. In contrast, AAFD_C_ and AAFD_L_ showed a moderately significant correlation, and CTR had a negligibly significant correlation with DG and DBP. Linear model equations were then generated for all significantly correlated parameters (Table 3).

**Table 3:** Coefficient of determination (r^2^) between each fetal parameter and the day of gestation (DG) and the day before parturition (DBP) with a linear regression equation.

Fetal parameters	Day of gestation (DG)		Day before parturition (DBP)	
	r^2^	Equation	r^2^	Equation
AAFD_C_	0.51*	DG = (mm – 0.54)/ 0.74	0.59*	DBP = (mm - 44.37)/ 0.79
AAFD_L_	0.50*	DG = (mm + 0.33)/ 0.78	0.56*	DBP = (mm - 45.79)/ 0.83
BPD	0.94*	DG = (mm + 11.93)/ 0.70	0.98*	DBP = (mm - 28.79)/ 0.72
CTR	0.01^o^	-	0.03 ^o^	-
ACA	0.89*	DG = (mm^2^ + 358)/ 108	0.92*	DBP = (mm^2^ - 2690)/ 109.40
GA	0.74*	DG = (mm^2^ + 328.50)/ 9.12	0.74*	DBP = (mm^2^ - 200.10)/ 9.14
ICS	0.75*	DG = (mm + 2.65)/ 0.09	0.75*	DBP = (mm - 2.48)/ 0.09

*P < 0.0001, not significant (*p* > 0.05) r^2^ = 0.90 to 1.00 (very high correlation), r^2^ = 0.50 to 0.70 (moderate correlation), and r^2^ = 0.00 to 0.30 (negligible correlation) (34), mm is the measured value.

### 3.4 Interrelationships among fetal parameters

This study investigated the correlations between various fetal parameters (BPD, ACA, GA, ICS, AAFD_C_, and AAFD_L_) throughout fetal development (Table 4). The relationship between BPD and ACA demonstrated the strongest correlation (r^2^ = 0.93, *p* < 0.001), indicating a close relationship in their developmental changes, whereas AAFD_C_ showed a negligible correlation with ICS and CTR (r^2^ = 0.23 and 0.18, respectively, *p* < 0.001), indicating the effect of thoracic development.

**Table 4:** Coefficient of regression (r^2^) between each fetal parameter.

**Fetal** **parameters**	**BPD**	**ICS**	**ACA**	**GA**	**AAFD**_**L**_	**AAFD**_**C**_
**ICS**	0.6580 *					
**ACA**	0.9307 *	0.7510 *				
**GA**	0.7177 *	0.6321 *	0.8199*			
**AAFD**_**L**_	0.0628	0.0170	0.0437	0.0155		
**AAFD**_**C**_	0.0061	0.2349 *	0.0106	0.0521	0.5274 *	
**CTR**	0.0852	0.0538	0.0056	0.0296	0.1055 *	0.1752 *

* Correlation is significant at the 0.001 level (*P* < 0.001), r^2^ = 0.90 to 1.00 (very high correlation), r^2^ = 0.50 to 0.70 (moderate correlation), and r^2^ = 0.00 to 0.30 (negligible correlation) (50).

## 4 Discussion

This study aimed to determine the development of AAFV and other fetal parameters in dogs by non-invasive techniques using ultrasonography, and we hypothesized that these parameters would be positively correlated during the pregnancy term, and that a strong interrelationship would exist among them, reflecting a synchronized and predictable growth pattern of fetuses. This study revealed that AAFD_C_ (max = 34.60 ± 7.89 mm) and AAFD_L_ (max = 33.51 ± 9.59 mm) increased during pregnancy, reached their maximum depth at week 6, and then decreased at the end of pregnancy (*p* < 0.05). Consistent with a previous study in dogs that measured actual volume, demonstrating a fetal fluid volume of approximately 122.28 mL (36). Like a previous study on canine and feline amniotic fluid and allantoic fluid, which found that actual amniotic volume peaked at mid-gestation before declining (36). Our study’s finding of a decrease in fetal fluid after mid-gestation, specifically AAFD_C_ and AAFD_L_, is consistent with previous studies in both canine and feline species (36). This decrease is attributed to the allantoic fluid’s relationship with kidney function, urachus duct occlusion, and urethra development. Conversely, AFV increased consistently throughout gestation, although its volume never surpassed that of allantoic fluid in late pregnancy (36, 37). However, this study also presented AAFD_C_ and AAFD_L,_ which showed a moderate correlation throughout gestation (weeks 3 to 8, *p* < 0.0001).

The fluid indicated the definitive kidney, which fully developed from the metanephros in the late term of canine pregnancy (37). Moreover, the volume of human amniotic fluid at any gestational stage reflects the dynamic water balance between fetus and mother. Imbalances of these regulations result in polyhydramnios or oligohydramnios, often due to abnormal fetal or maternal disorders (38, 39). Further investigation is therefore needed to explore the practical application of this finding to perinatal pregnancy evaluation in veterinary practice, as this correlation has not yet been reported. Future research should specifically focus on monitoring intestinal and renal development to provide a more comprehensive understanding of AAFV development.

The changes in fetal parameters throughout gestation reflect continuous growth and development (*p* < 0.0001). Specifically, BPD indicates head growth, while ACA and GA demonstrate abdominal and internal organ expansion. ICS and CTR signify thoracic cavity enlargement, correlating with the development of intrathoracic organs (1, 40, 41). Moreover, this study reveals divergent trends for fetal BPD, ACA, GA, and ICS, which reflect fetal head, abdominal, and thoracic growth and organ dimensions. This suggests a lack of a correlation between the growth of these structures and AAFD_C_ and AAFD_L_. This is further supported by the negligible correlation observed in our statistical analysis, especially concerning thoracic cavity enlargement (*p* < 0.001). Our results are consistent with human research showing no direct relationship between fetal fluid volume and embryonic or organ size (38, 42, 43). However, the volume of amniotic fluid can be used for assessment of lung, gastrointestinal, and urinary maturation and maldevelopment in humans (44–49) and cats (37).

## 5 Conclusion

This study is the first to report the development patterns of amniotic fluid volume in dogs, using a non-invasive ultrasonographic approach. It also elucidates the relationship between amniotic fluid parameters (AAFD_C_ and AAFD_L_) and other relevant factors. These findings propose the establishment of AAFD_C_ and AAFD_L_ as novel diagnostic markers for evaluating canine fetal well-being in clinical practice. It is crucial to note that these markers are intended to be complementary tools that provide additional and supportive data to enhance the overall accuracy of perinatal evaluation.

## Data Availability

The raw data supporting the conclusions of this article will be made available by the authors, without undue reservation.

## Glossary

LH: Luteinizing hormone
ICC: Inner chorionic cavity
OUD: Outer uterine diameter
CRL: Crown rump length
BPD: Biparietal diameter
DPTV: Deep portion of diencephalo-telencephalic vesicle
AF: Amniotic fluid
AFV: Amniotic fluid volume
AAF: Allantoamniotic fluid
AAFV: Allantoamniotic fluid volume
AAFD_C_: Allantoamniotic fluid depth by cross-sectional view
AAFD_L_: Allantoamniotic fluid depth by longitudinal view
SDVP: Single deepest vertical pocket
AI: Artificial insemination
SDP: Single deepest pocket
DVP: Deepest vertical pocket
CTR: Cardio-thoracic ratio
ACA: Abdominal cross-sectional area
GA: Gastric area
ICS: Intercostal space
DG: Day of gestation
DBP: Day before parturition
Wk: Week
